# The Use of Systemic Inflammatory Markers From Routine Blood Tests in Predicting Preeclampsia and the Impact of Age on Marker Levels

**DOI:** 10.7759/cureus.35836

**Published:** 2023-03-06

**Authors:** Giorgi Maziashvili, Kathereene Juliana, Vivek Siva Subramania Pillai Kanimozhi, Giorgi Javakhishvili, Vakhtang Gurabanidze, Tinatin Gagua, Tamar Maziashvili, Khatuna Lomouri

**Affiliations:** 1 Faculty of Medicine, Tbilisi State Medical University, Tbilisi, GEO; 2 Obstetrics and Gynecology, Gagua Clinic, Tbilisi, GEO; 3 Faculty of Health Sciences, Bahcesehir University (BAU) International University, Batumi, GEO; 4 Neonatology, Tbilisi State Medical University, Tbilisi, GEO

**Keywords:** systemic inflammatory markers, cbc, first trimester of pregnancy, pregnancy, hypertension, inflammation, preeclampsia

## Abstract

Our study aimed to investigate the relationship between preeclampsia (PE) and blood levels of neutrophil-to-lymphocyte ratio (NLR), monocyte-to-lymphocyte ratio (MLR), platelet-to-lymphocyte ratio (PLR), and systemic immune-inflammatory index (SII) in the first trimester of pregnancy. In addition to examining the potential correlation between these inflammatory markers and PE, we aimed to compare the levels based on age to determine whether there are potential age-related differences in marker levels.

Over a six-month period, we reviewed the complete blood count (CBC) analysis results of 126 subjects, where 63 patients had a documented history of PE and 63 were healthy pregnant females. We found that age had no statistically significant effect on NLR, MLR, or SII levels, but there was a statistically significant difference in PLR levels between the 18-25 and 26-35 age groups.

The study also revealed that the MLR and PLR in the 18-25 age group of preeclampsia patients were statistically significantly lower than those of healthy patients, whereas the PLR and SII in the 26-35 age group of preeclampsia patients were statistically significantly higher than those of healthy patients.

The results suggest that systemic inflammatory response (SIR) markers may be able to predict the development of preeclampsia. The study also emphasized the importance of taking age into account, specifically the 18-25 and 26-35 age groups, when assessing the risk of preeclampsia. Further research is needed however to corroborate existing findings and determine the importance of the examined inflammatory markers in the diagnosis of PE.

## Introduction

Systemic inflammation is a condition where the body’s immune system is activated in response to a range of stimuli, including infections, stress, and physical trauma [1]. As a result, the body produces inflammatory mediators such as cytokines, which cause the release of an array of substances known as inflammatory markers [2]. These inflammatory markers can be used to detect the presence of systemic inflammation and can provide an early indication of potential diseases or conditions. Studies have suggested that maternal systemic inflammation is associated with increased susceptibility to preeclampsia (PE) [3,4]. PE is a hypertensive disorder of pregnancy that can lead to serious maternal and fetal complications [5]. It is characterized by hypertension along with proteinuria and can lead to the death of the mother and the child [6]. PE is currently diagnosed based on clinical observation and laboratory evaluation, and early identification still remains a challenge due to its insidious onset. Because of that, the identification of biomarkers of systemic inflammation as early indicators of PE is of great interest.

The role of systemic inflammatory markers in the development of PE is still not fully understood. However, several studies have suggested that an inflammatory response in the mother’s body, which causes changes in the maternal immune system, is involved in the pathogenesis of PE [7,8]. Nuclear factor kappa B (NF-κB), myeloperoxidase (MPO), and serum interleukin 6 (IL-6) are among the systemic inflammatory markers that have been investigated as potential predictors of PE. NF-κB is a transcription factor that is involved in the regulation of the immune response [9]. Studies have shown that NF-κB is highly expressed in preeclamptic females as compared to normotensive controls [9]. Similarly, MPO, an enzyme produced by neutrophils, has been found to be elevated in preeclamptic females compared to controls [10]. Furthermore, studies have demonstrated that IL-6 is significantly increased in pregnant females with PE [11]. Although measuring these potential markers can be used to predict the development of PE, they are not included in first-trimester pregnancy screening and are therefore an impractical solution due to financial constraints, especially for patients in developing nations such as Georgia.

Markers of systemic inflammation that can be calculated from complete blood count (CBC) analysis have been proposed as potential early predictors of PE. These biomarkers are neutrophil-to-lymphocyte ratio (NLR), platelet-to-lymphocyte ratio (PLR), monocyte-to-lymphocyte ratio (MLR), and systemic immune-inflammatory index (SII). Elevated levels of these markers in the first and second trimesters of pregnancy have been associated with an increased risk of developing PE. For example, a study by Lamarca et al. found that an NLR of more than 2.5 in the first trimester was associated with a 3.5-fold increased risk of developing preeclampsia [11]. Additionally, findings by Kang et al. suggested that the NLR had a great predictive value in severe preeclampsia patients [12].

Most recently, Oğlak et al. stated that the first-trimester NLR and PLR values were useful in predicting PE [13]. CBC analysis is included in Georgia’s national health government program; therefore, conducting simple calculations to determine the NLR, MLR, PLR, or SII from the routinely performed test could be a very helpful tool to determine which individuals would benefit from early therapeutic intervention without any extra cost associated with the screening. Although the abovementioned studies showed the potential correlation of increased markers of systemic inflammation with early identification of PE, results have been conflicting and require further evaluation. In addition to investigating the correlation between these inflammatory markers and PE, our study aimed to examine the levels of NLR, MLR, PLR, and SII between different age groups. We believe that this additional aim could provide valuable insights into the identification and management of PE across various age groups. It is noteworthy that none of the prior studies have addressed this research question, and we are the first to investigate the potential age-related differences in these markers.

## Materials and methods

The retrospective case-control study was conducted at Gagua Clinic, Tbilisi, Georgia. The study was approved by the clinic’s ethics committee. The CBC analysis results of 63 patients with a documented history of preeclampsia during the six-month period (January-July 2022) were reviewed. The control group consisted of randomly selected 63 healthy normotensive pregnant females who gave birth after 37 weeks of gestation. All preeclamptic patients included in the study were diagnosed after 20 weeks of gestation with hypertension of systolic blood pressure of >140 mmHg and diastolic blood pressure of >90 mmHg and the onset of proteinuria of >0.3 g (24-hour urine), urine dipstick protein of 1+, or end-organ damage. The control group consisted of females who gave birth between 37 and 41 weeks of gestation and had no documented history of complications during the pregnancy.

The clinical data collected from the electronic medical records included first-trimester CBC analysis results, maternal age, and history of preeclampsia. CBC analysis results taken during the first trimester (7-14 weeks) of pregnancy were analyzed to determine the mean levels of NLR, MLR, PLR, and SII. If more than one CBC test was performed during the first trimester, the one closest to seven weeks of pregnancy was used for statistical analysis. The NLR is calculated by dividing the absolute neutrophil count by the absolute lymphocyte count. The MLR is calculated by dividing the absolute monocyte count by the absolute lymphocyte count. The PLR is calculated by dividing the absolute platelet count by the absolute lymphocyte count. The systemic immune-inflammatory index (SII) is calculated by multiplying the platelet count and neutrophil count and then dividing the result by the lymphocyte count.

Patients were divided into three age groups: 18-25 years, 26-35 years, and 36+ years. Statistical Package for Social Sciences (SPSS) version 26 (IBM SPSS Statistics, Armonk, NY) and Minitab version 17 (Minitab, LLC, State College, PA) were used for statistical analysis. The descriptive analysis is followed by the independent sample t-test and one-way analysis of variance (ANOVA) analysis in order to determine the difference in increased markers of systemic inflammation (NLR, MLR, PLR, and SII) between the patients with preeclampsia and healthy control group in three age groups. The relative risks will be estimated with 95% confidence intervals (CIs). A statistically significant difference is considered as a p-value of less than 0.05.

## Results

Table 1 shows the distribution of age of the two study groups. The majority (49.2%) of the preeclampsia patients and the majority (54%) of the control group are 26-35 years old.

**Table 1:** Age distribution of the preeclampsia group and the control group

Group of patients	Age group	Frequency	Percent
Preeclampsia	18-25	16	25.4
	26-35	31	49.2
	36+	16	25.4
	Total	63	100
Control	18-25	23	36.5
	26-35	34	54
	36+	6	9.5
	Total	63	100

Table 2 illustrates that the NLR of preeclampsia patients ranged from 0.18 to 6.59, with a mean of 3.22±1.17, while the NLR of the control group ranged from 1.51 to 6.80, with a mean of 3.04±1.06. Similarly, the MLR of preeclampsia patients ranged from 0.03 to 0.47, with a mean of 0.24±0.09, and for the control group, the values ranged from 0.14 and 0.57, with a mean of 0.29±0.10. The PLR value in preeclampsia patients ranged from 7.60 to 274.10, with a mean of 132.01±42.63, while the control group had a range of 64.86-225.42, with a mean of 128.65±33.44. Lastly, SII levels in the patients with preeclampsia ranged from 43.33 to 1996.94, with a mean of 885.67±388.19, and the levels of the control group ranged from 332.28 to 1482.00, with a mean of 780.68±277.99.

**Table 2:** Descriptive statistics of the markers of the preeclampsia group and the control group NLR, neutrophil-to-lymphocyte ratio; MLR, monocyte-to-lymphocyte ratio; PLR, platelet-to-lymphocyte ratio; SII, systemic immune-inflammatory index; SD, standard deviation

Group of patients	Marker	Minimum	Maximum	Mean	SD
Preeclampsia	NLR	0.18	6.59	3.22	1.17
	MLR	0.03	0.47	0.24	0.09
	PLR	7.60	274.10	132.01	42.63
	SII	43.33	1996.94	885.67	388.19
Control	NLR	1.51	6.80	3.04	1.06
	MLR	0.14	0.57	0.29	0.10
	PLR	64.86	225.42	128.65	33.44
	SII	332.28	1482.00	780.68	277.99

**Figure 1:**
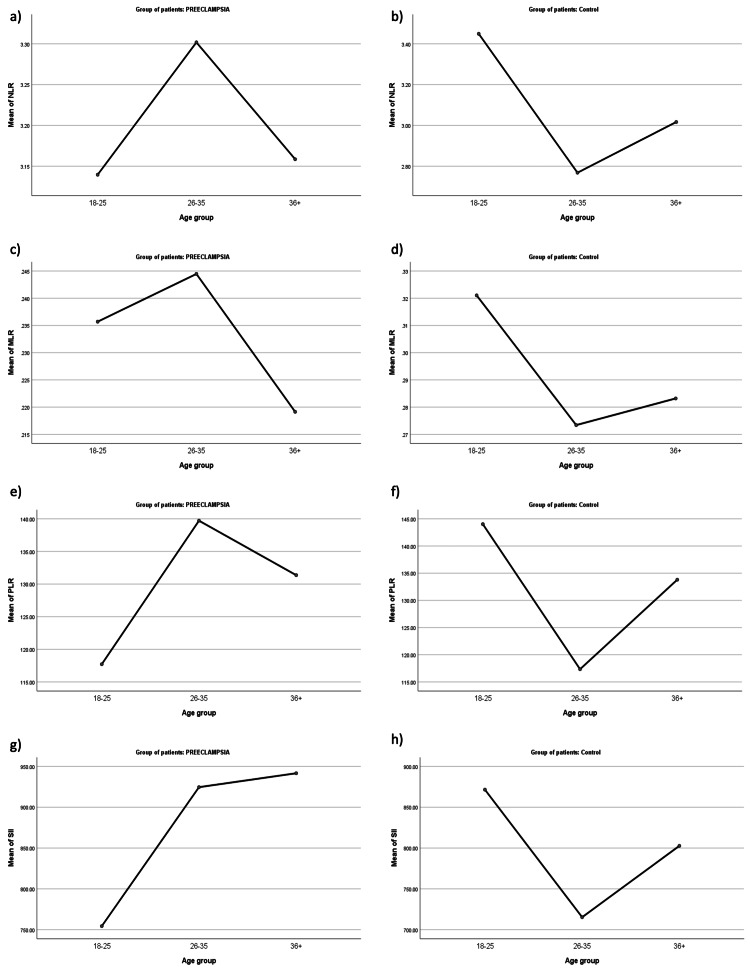
Mean NLR, MLR, PLR, and SII of preeclampsia and healthy patients across three age groups NLR, neutrophil-to-lymphocyte ratio; MLR, monocyte-to-lymphocyte ratio; PLR, platelet-to-lymphocyte ratio; SII, systemic immune-inflammatory index

One-way ANOVA analysis was performed to determine the influence of age group (independent variable) on the different marker levels (dependent variable) of preeclampsia and control groups. Figure 1 demonstrates that the 26-35 age group has the highest levels of NLR, MLR, and PLR among preeclampsia patients. However, the one-way ANOVA results indicate that the differences between the age groups are not statistically significant. The 18-25 age group has the highest mean NLR, MLR, and SII in the control group, while the 36+ age group has the lowest mean NLR, MLR, and PLR. The one-way ANOVA results demonstrate that the differences between these age groups are not statistically significant, with the exception of the PLR comparison. This comparison reveals a statistically significant difference between the 18-25 age group and the 26-35 age group (p=0.010). This indicates that age does not have a major impact on NLR, MLR, and SII levels, although there is a statistically significant difference in PLR levels between the 18-25 and 26-35 age groups.

Two sample independent t-tests were performed to evaluate the differences of markers of systemic inflammation between the preeclampsia group and the control group. As shown in Table 3, the MLR of the preeclampsia group (M=0.24; standard deviation {SD}=0.09) is lower than the control group (M=0.29; SD=0.10). The results of two sample independent t-tests indicate that the difference is statistically significant (t(124)=3.35; p=0.001). The rest of the markers show no statistically significant difference between the two groups (p<0.05).

**Table 3:** Results of the independent t-test for markers between the preeclampsia group and the control group NLR, neutrophil-to-lymphocyte ratio; MLR, monocyte-to-lymphocyte ratio; PLR, platelet-to-lymphocyte ratio; SII, systemic immune-inflammatory index; SD, standard deviation

Marker	Group of patients	Mean	SD	T-value	P-value
NLR	Preeclampsia	3.22	1.17	0.925	0.357
	Control	3.04	1.06		
MLR	Preeclampsia	0.24	0.09	3.35	0.001
	Control	0.29	0.10		
PLR	Preeclampsia	132.01	42.63	0.49	0.624
	Control	128.65	33.44		
SII	Preeclampsia	885.67	388.19	1.74	0.083
	Control	780.68	277.99		

**Figure 2:**
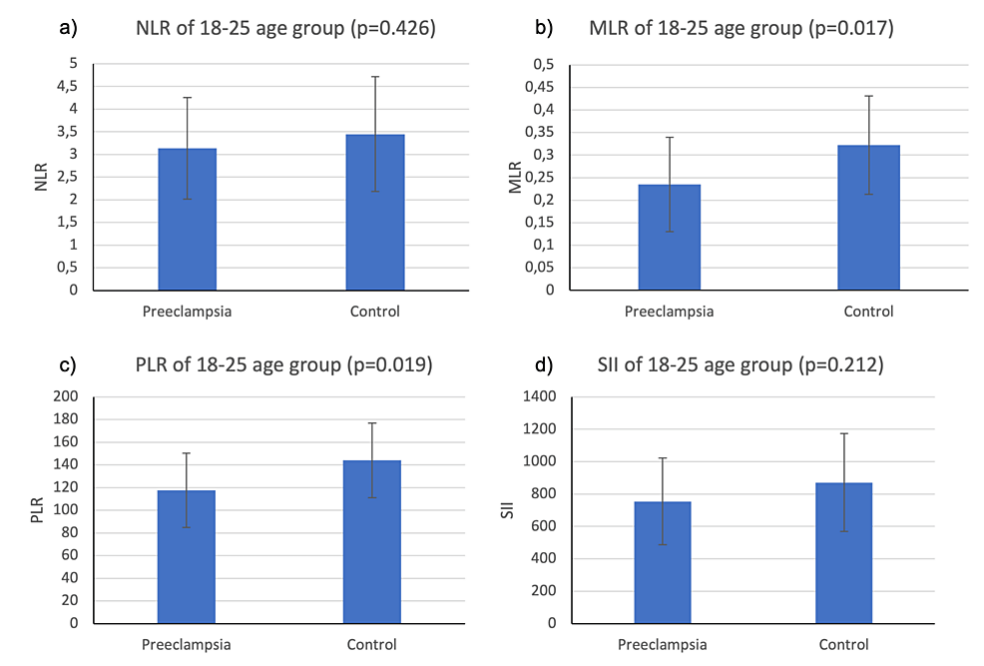
Mean NLR, MLR, PLR, and SII values for preeclampsia and healthy patients in the 18-25 age group The vertical bar on the graph shows the standard deviation NLR, neutrophil-to-lymphocyte ratio; MLR, monocyte-to-lymphocyte ratio; PLR, platelet-to-lymphocyte ratio; SII, systemic immune-inflammatory index

**Figure 3:**
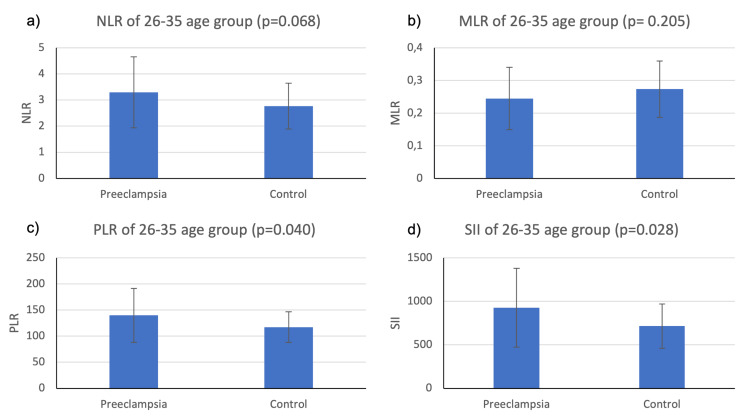
Mean NLR, MLR, PLR, and SII values for preeclampsia and healthy patients in the 26-35 age group The vertical bar on the graph shows the standard deviation NLR, neutrophil-to-lymphocyte ratio; MLR, monocyte-to-lymphocyte ratio; PLR, platelet-to-lymphocyte ratio; SII, systemic immune-inflammatory index

**Figure 4:**
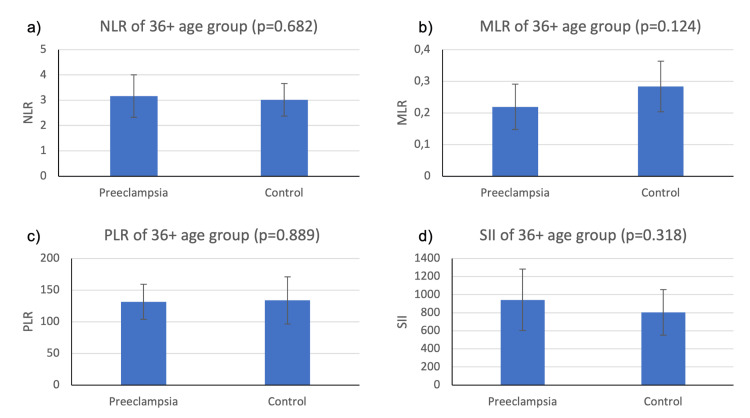
Mean NLR, MLR, PLR, and SII values for preeclampsia and healthy patients in the 36+ age group The vertical bar on the graph shows the standard deviation NLR, neutrophil-to-lymphocyte ratio; MLR, monocyte-to-lymphocyte ratio; PLR, platelet-to-lymphocyte ratio; SII, systemic immune-inflammatory index

We also evaluated the differences of markers of systemic inflammation between the preeclampsia group and the control group under each age category using two sample independent t-tests. The results of the comparison of the preeclampsia and control groups, as shown in Figures 2-4, indicate varying levels of significance.

Figure 2 reveals that in the 18-25 age group, the NLR of the preeclampsia group (M=3.14; SD=1.12) was lower than the control group (M=3.45; SD=1.27), but the difference was not statistically significant as determined by the two sample independent t-tests (t(34)=0.81; p=0.426). The MLR of the preeclampsia group (M=0.24; SD=0.11) was lower than the control group (M=0.32; SD=0.11), and the difference was statistically significant (t(33)=2.51; p=0.017). The PLR of the preeclampsia group (M=117.70; SD=32.70) was lower than the control group (M=144; SD=32.80), and the difference was statistically significant (t(32)=2.47; p=0.019). The SII of the preeclampsia group (M=754; SD=268) was lower than the control group (M=871; SD=303), but the difference was not significant (t(34)=1.27; p=0.212).

As seen in Figure 3, in the 26-35 age group, the NLR of the preeclampsia group (M=3.30; SD=1.36) was higher than the control group (M=2.77; SD=0.88), but the difference was not statistically significant (t(50)=1.86; p=0.068). The MLR of the preeclampsia group (M=0.24; SD=0.09) was lower than the control group (M=0.27; SD=0.09), and the difference was not statistically significant (t(60)=1.28; p=0.205). The PLR of the preeclampsia group (M=139.70; SD=51.70) was higher than the control group (M=117.30; SD=29.50), and the difference was statistically significant (t(46)=2.12; p=0.040). The SII of the preeclampsia group (M=925; SD=453) was higher than the control group (M=715; SD=254), and the difference was statistically significant (t(46)=2.27; p=0.028).

Figure 4 shows the results of the 36+ age group, which showed that the NLR of the preeclampsia group (M=3.16; SD=0.84) was higher than the control group (M=3.02; SD=0.65), but the difference was not statistically significant (t(11)=0.42; p=0.682). The MLR of the preeclampsia group (M=0.219, SD=0.07) was lower than the control group (M=0.28; SD=0.08), and the difference was not statistically significant (t(8)=1.72; p=0.124). The PLR of the preeclampsia group (M=131.40; SD=27.60) was lower than the control group (M=133.80; SD=37.40), and the difference was not statistically significant (t(7)=0.15; p=0.889). The SII of the preeclampsia group (M=942; SD=340) was higher than the control group (M=803; SD=252), but the difference was not statistically significant (t(12)=1.04; p=0.318).

Overall, the comparison between the preeclampsia and control groups revealed varying levels of significance. The MLR and PLR in the 18-25 age group of the PE patients were significantly lower than the healthy patients, while the PLR and SII in the 26-35 age group of the preeclampsia patients were significantly higher than the healthy patients. The NLR of the preeclampsia group was both lower and higher than the control group, but neither difference demonstrated statistical significance.

## Discussion

Preeclampsia is a major complication of pregnancy that is a leading cause of maternal and fetal morbidity and mortality. The early prediction of preeclampsia is of paramount importance for timely management, which can help reduce maternal and perinatal mortality and morbidity. Recent studies have revealed that conducting simple calculations to determine the NLR, MLR, PLR, or SII from the routinely performed test could be a very helpful tool for the early identification of PE.

Oğlak et al. discovered that having an NLR greater than 2.5 in the first trimester was related to a 3.5-fold increased chance of developing preeclampsia [13]. In our study, the mean value of NLR among patients with preeclampsia was 3.22 with a standard deviation of 1.17. Findings by Kang et al. suggested that NLR levels were higher among patients with PE [12]. However, there was no statistically significant difference between PE patients and healthy pregnant females in our study. Our findings matched those of Yavuzcan et al., who found no statistically significant difference in NLR levels between PE patients and healthy pregnant females [14].

Oğlak et al. reported that the first-trimester NLR and PLR values were helpful for predicting PE [13]. We did find a statistically significant difference in PLR values in the 18-25 age group, though a higher value was observed in healthy pregnant females. Data reveals that the 26-35 age group showed statistically significantly higher PLR levels in PE patients. It is important to note that when comparing two groups without considering age, the only statistically significant difference we observed was in the MLR levels (p=0.01), where healthy pregnant females had higher mean value than PE patients. Tarca et al. found that SII had a pooled sensitivity of 0.87 and specificity of 0.79 for the prediction of preeclampsia [15]. When we compared the SII values of the two patient groups without respect to their ages, we found no statistically significant difference. Nevertheless, SII levels in preeclampsia patients aged 26-35 were statistically significant. This data is important, as it suggests that age should be taken into account when assessing the risk of preeclampsia, particularly in the 18-25 and 26-35 age groups.

It should also be mentioned that our study had a limitation in that age alone cannot be the sole factor considered when assessing the risk of preeclampsia. Other relevant factors, such as a patient’s lifestyle and medical history, including chronic conditions, past pregnancies and deliveries, daily medications, and other instances that may potentially alter the inflammatory markers in blood, must also be carefully evaluated. Our study demonstrates the potential for using certain systemic inflammatory response (SIR) markers to predict the development of preeclampsia. Further studies should be conducted to confirm the role of those SIR markers that showed statistically significant value; in the diagnosis of PE and to strengthen the current findings, additional research is required.

## Conclusions

In conclusion, this study suggests that certain SIR markers have the potential to predict the development of preeclampsia. Results also suggest that age should be taken into account when assessing the risk of preeclampsia, particularly in the 18-25 and 26-35 age groups. However, additional research is required to confirm these findings and to establish the role of SIR markers in the diagnosis of preeclampsia.
